# LMP1 and LMP2A are potential prognostic markers of extranodal NK/T-cell lymphoma, nasal type (ENKTL)

**DOI:** 10.1186/1746-1596-7-178

**Published:** 2012-12-13

**Authors:** Yuan Mao, Da-Wei Zhang, Huijun Zhu, Hong Lin, Lin Xiong, Qing Cao, Ying Liu, Qing-Dong Li, Jia-Ren Xu, Lin-Feng Xu, Ren-Jie Chen

**Affiliations:** 1 Department of Otolaryngology-Head and Neck Surgery, Jiangsu Provincial Hospital, No.65 Jiangsu Road, Nanjing, 210029, China; 2 Department of Otolaryngology-Head and Neck Surgery, The Second Affiliated Hospital of Nanjing Medical University, No.121 Jiang jia yuan, Nanjing 210011, China; 3 Department of Pathology, Affiliated Hospital of Nantong University, No.20 Xisi Road, Nantong, 226001, China; 4 Jiangsu Provincial Blood Center, No.179 Longpan Road, Nanjing, 210042, China; 5 Department of Pathology, The Second Affiliated Hospital of Nanjing Medical University, No.121 Jiang jia yuan, Nanjing, 210011, China; 6 Department of Hematology and Oncology, Jiangsu Provincial Hospital, No.65 Jiangsu Road, Nanjing 210029, China; 7 Department of General Surgery, The Second Affiliated Hospital of Nanjing Medical University, No.121 Jiang jia yuan, Nanjing 210011, China

**Keywords:** LMP1, LMP2A, EBV, ENKTL, Prognosis, Tumor marker

## Abstract

**Background:**

Latent membrane protein (LMP) 1 and LMP2A encoded by Epstein-Barr virus (EBV) are associated with the development of malignancies, but their expression in extranodal NK/T-cell lymphoma, nasal type (ENKTL) and the relationship with clinical characteristics of this disease remain poorly understood. In the present study, we examined the expression of LMP1 and LMP2A in ENKTL, and investigated the correlations between LMP1 and LMP2A expression with clinicopathological characteristics of ENKTL patients.

**Methods:**

Paraffin sections of surgically removed samples from 16 ENKTL patients were analyzed by immunohistochemistry and the related clinicopathological data were collected and analyzed.

**Results:**

Elevated expression (immunohistochemistry score ≥ 4) of LMP1 and LMP2A was detected in the tumor cells of ENKTL. High LMP1 expression was associated with positive B symptoms (p = 0.012), while high LMP2A expression was related to gender (p = 0.029). The expression of both LMP1 and LMP2A showed significant correlations with patients’ overall survival (p = 0.049, p = 0.036).

**Conclusion:**

LMP1 and LMP2A may be prognostic indicators of survival in patients with ENKTL.

**Virtual slides:**

http://www.diagnosticpathology.diagnomx.eu/vs/2443352538545899

## Introduction

Extranodal NK/T-cell lymphoma, nasal type (ENKTL) is recognized as a distinct clinical pathological entity of non-Hodgkin’s lymphoma, and develops more frequently in East Asia and Central America than in the West [1,2]. ENKTL was previously described as lethal midline granuloma or midline reticulosis, which most commonly invades the nasal cavity and other mucosal sites of the upper aerodigestive tract. Although ENKTL can be diagnosed at early stage, it is characterized by a strongly aggressive lymphoma in advanced stage and resistance to different treatments [3]. Currently, ENKTL is mainly treated by concurrent chemoradiotherapy [4-6], but the overall survival is frustratingly poor [7]. Among the risk factors of ENKTL development, Epstein-Barr virus (EBV) latent infection has been shown to play an important role, and ENKTL is closely associated with EBV infection [8-11].

EBV is a member of the g-herpes- virus family which approximately infects 90% of the adult population in the world. A variety of molecules are involved in EBV latent infection, including EBV-encoded nuclear antigens (EBNAs); EBNA leader protein (EBNA-LP); latent membrane protein (LMP) 1, LMP2A, and LMP2B; and EBV encoded RNAs (EBERs) EBER1 and EBER2 [12]. Among these, LMP1 is essential for EBV-mediated growth transformation of infected cells, and the C-terminal region of LMP1 protein can regulate a variety of cellular signaling pathways such as TNF receptor, NF-κB and JAK/STAT to regulate the proliferation, immortalization, and invasion of lymphoma cells [13-15]. Therefore, LMP1 has been suggested to have oncogenic effects in the development and progression of EBV-related lymphomas. On the other hand, LMP2A is one of the most commonly present EBV encoding proteins and is widely expressed in EBV-infected cells within the infected human host and EBV-associated malignancies. LMP2A is the only one membrane protein expressed in the reservoir of circulating, latently EBV-infected resting B-cells [16]. LMP2A contributes to malignant transformation by intervening with multiple signaling pathways, especially the cell cycle and apoptotic pathways, thus plays important role in viral latency and tumorigenesis [17,18]. The ability of LMP2A to influence the balance of survival factors in B lymphocytes could be functionally important in Burkitt lymphoma and LMP2A expression in EBV-infected B cells may lead to the induction and maintenance of an activated, proliferative state that could ultimately result in the development of Hodgkin lymphoma [19,20]. However, the correlation between the expression of LMP1 and LMP2A in ENKTL and patient prognosis is poorly understood.

In this study, we examined the expression profiles of LMP1 and LMP2A in ENKTL patients by immunohistochemistry analysis. Moreover, we analyzed the correlation of LMP1 and LMP2A expression with the clinicopathologic features and outcomes of ENKTL. Our results suggest that LMP1 and LMP2A are potential biomarkers for the diagnosis and prognosis of patients with ENKTL.

## Materials and methods

### Patients and diagnosis

Total 16 cases of ENKTL were enrolled in this study who were diagnosed at Department of Pathology, the Affiliated Hospital of Nantong University between 2005 to 2011. All 16 cases were histologically confirmed, with complete clinical information and follow-up data. The pathologic diagnosis and classification of ENKTL was based on the criteria described previously [1,21]: 1) current World Health Organization classification; 2) the histologic characteristics of ENKTL; 3) EBER expression by in situ hybridization; 4) the expression of CD3 (T cell markers) and CD56 by immunohistochemistry; 5) the absence of B cell phenotype with CD20. Cases which were negative for CD56 while positive for both cytotoxic markers and EBER were also categorized as ENKTL. Written informed consent was obtained from the patient for publication of this report and any accompanying images. Study protocol was approved by the Ethics Committee of Jiangsu Province Official Hospital.

### Data extraction

Clinical data of 16 cases were obtained from hospital medical records, which included age distribution, patient sex ratio, initial symptoms and signs, primary sites, Ann Arbor stage, lactate dehydrogenase (LDH) level, the presence of B symptoms, international prognostic index (IPI), therapy strategies (surgery, chemotherapy or radiotherapy), response to treatment and survival status.

### Histologic analysis

ENKTL tissue sections were formalin fixed, paraffin-embedded and H&E-stained. The histopathological features of the cases were evaluated independently by two investigators.

### In *situ* hybridization

EBERs in *situ* hybridization was performed on formalin-fixed paraffin-embedded tissue sections on 3-aminopropyltriethoxy-silane (Sigma, St. Louis, MO, USA) treated slides using fluorescein isothiocyanate-conjugated EBERs oligonucleotides as probes. Evaluation of hybridized sites was detected by anti-fluorescein isothiocyanate antibody labeled with alkaline phosphatase (Dako, Cytomation, Glostrup, Denmark), according to the manufacturer’s protocols. The control procedures, including positive and negative control sections, were conducted simultaneously.

### Immunohistochemistry

Paraffin tissue sections were deparaffinized in 100% xylene and rehydrated in graded ethanol solutions. After antigen retrieval the sections were immersed in a 0.3% hydrogen peroxide solution for 15 min to block endogenous peroxidase activity. Nonspecific binding was blocked by incubation with 5% goat serum in TBS for 30 min. Tissue sections were then incubated with monoclonal antibodies anti-CD3 (Novocastra, Newcastle, UK), anti-CD20 (Dako), anti-CD56 (Dako), anti-TIA-1 (Abcam, Cambridge, UK), anti-LMP1 (Abcam) and anti-LMP2A (Abcam), followed by incubation with EnVision horseradish peroxidase complex (DAKO). Negative controls were included by the replacement of the primary antibody with phosphate-buffered saline. Four fields in each slide were randomly selected and counted, the percentage of positive staining was determined using immunohistochemistry score (IHS) [22]. When a conclusion differed, the final decision was made by consensus. Results were analyzed according to the method described previously [23]. Briefly, IHS was determined by the evaluation of both staining density and intensity. The percentage of positive tumor cells was scored as follows: 1 (0-10% positive cells), 2 (11-50% positive cells), 3 (51-80% positive cells), 4 (81-100% positive cells); and the intensity of staining was scored as follows: 0 (negative), 1 (weakly positive), 2 (moderately positive), and 3 (strongly positive). Multiplication of the intensity and the percentage scores gave rise to the ultimate immunoreactivity score: samples with a sum score below 3 (IHS ≤ 3) were judged as low protein expression, and those with a sum score above 4 (IHS ≥ 4) as high protein expression.

### Statistical analysis

Statistical analyses were carried out by using STATA Version 12.0 (Stata Corporation, College Station, TX) and SPSS 18.0 statistic software (SPSS Inc, Chicago, IL). The correlation of LMP1 and LMP2A expression with clinicopathological features of ENKTL was analyzed by chi-square test. Overall survival rate was estimated by Kaplan-Meier method and statistical significance was assessed by the log-rank test. P < 0.05 was considered as statistical significance.

## Results

### Clinical features of ENKTL

The main clinicopathologic characteristics of patients with ENKTL were shown in Table 1. All ENKTL patients showed positive staining for CD3, TIA-1 and EBER-1 and negative staining for CD20. CD56 expression was present in 43.8% of 16 cases. There was a preponderance of male to female patients with a ratio of 13:3. The median age at diagnosis was 49.6 years (range 25–71 years). Among the 16 cases, 11 were in the nasal cavity, two in the oropharynx and/or palate, two in the larynx, and one in nasopharynx. Nasal obstruction was the most common initial symptom. According to the Ann Arbor stage of lymphoma, five patients were in stage I, nine in stage II and two in stage III of ENKTL. Five patients showed high LDH expression, and eight patients encountered B symptoms, including fever, night sweats, and weight loss. Furthermore, the IPI was used to predict the survival of patients with ENKTL. Most of the patients (11 of 16) presented low to intermediate risk, and the other five patients exhibited high to intermediate risk. Of all 16 ENKTL patients, six patients received chemotherapy, four patients received a combination of surgery and chemotherapy, four patients received a combination of chemotherapy and radiotherapy, and two patients received surgery alone. Seven patients experienced complete remission (CR) (43.8%) and eight patients experienced partial remission (PR) (50.0%), while one patient had no response (NR) (6.2%).

**Table 1:** Clinical and histologic features of 16 patients with ENKTL

**Patient no.**	**Age**	**Sex**	**Primary sites**	**Ann Arbor stage**	**LDH level**	**B symptoms**	**IPI**	**Therapy**	**Response to treatment**	**LMP1 expression (IHS)**	**LMP2A expression (IHS)**
1	36	M	Nasal cavity	II	Normal	Positive	LI	CHOP + FLAG	PR	6	6
2	36	M	Oropharynx	III	High	Positive	HI	CHOP	PR	4	2
3	57	M	Nasal cavity	II	Normal	Negative	HI	Surgery + CHOP	CR	2	1
4	55	M	Larynx	I	Normal	Positive	LI	Surgery + CHOP	PR	8	1
5	49	F	Nasal cavity	II	High	Negative	L	Surgery	NR	1	4
6	51	M	Nasal cavity	I	High	Negative	LI	CHOP + Radiotherapy	PR	2	1
7	67	M	Nasal cavity	II	Normal	Positive	HI	CHOP + GDP + CVAD	CR	9	4
8	25	M	Oropharynx, palate	II	Normal	Negative	L	CHOPE + ICE + Radiotherapy	CR	1	9
9	71	M	Nasal cavity	I	High	Positive	LI	CHOP + SMILE	CR	4	2
10	36	M	Nasal cavity	II	Normal	Negative	L	CHOP	PR	6	1
11	63	M	Nasal cavity	I	Normal	Negative	L	CEVP + Radiotherapy	PR	2	1
12	56	M	Nasal cavity	II	Normal	Positive	LI	Surgery + CHOP	PR	3	2
13	66	F	Larynx	II	High	Positive	HI	Surgery	CR	6	8
14	47	M	Nasopharynx	I	Normal	Negative	L	VEPA + Radiotherapy	CR	8	1
15	51	F	Nasal cavity	III	Normal	Positive	HI	DICE + SMILE	PR	9	9
16	27	M	Nasal cavity	II	Normal	Negative	LI	Surgery + CHOP	CR	3	6

LDH: lactate dehydrogenase; IPI: international prognostic index; IHS: immunohistochemistry score; LMP1: latent membrane protein 1; LMP2A: latent membrane protein 2A; L: low risk; LI: low-intermiediate risk; HI: high-intermiediate risk; CR: complete remission; PR: partial remission; NR: no response; CHOP: cyclophosphamide, hydroxydaunomycin, oncovin, prednisone; FLAG: fludarabine, arabinosylcytosine, granulocyte colony-stimulating factor; GDP: gemcitabine, dexamethasone, cisplatin; CVAD: cyclophosphamide, vindesine sulfate, adriamycin, cisplatin; CHOPE: cyclophosphamide, hydroxydaunomycin, oncovin, prednisone, etoposide; ICE: ifosfamide, cisplatin, etoposide; SMILE: steroid, methotrexate, ifosfamide, l-asparaginase, etoposide; CEVP: cyclophosphamide, epirubicin, vincristine, prednisone; VEPA: vincristine, cyclophosphamide, doxorubicin, predonisolone; DICE: dexamethasone, ifosfamide, cisplatin, etoposide.

### Expression of LMP1 and LMP2A in ENKTL

**Figure 1:**
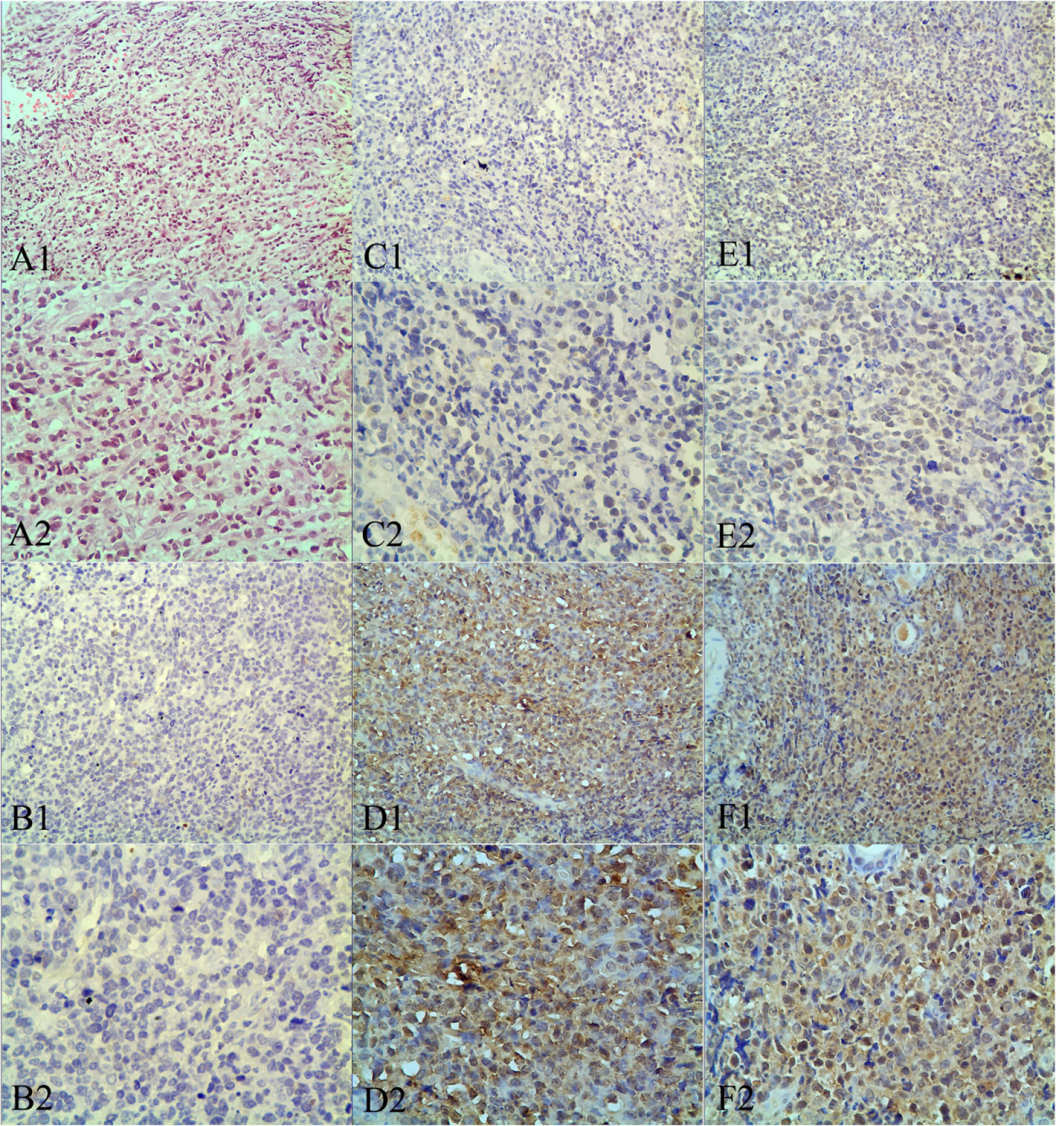
**Immunohistochemical staining of LMP1 and LMP2A in clinical tissue samples of ENKTL.** (**A1** and **A2**) Hematoxylin-eosin staining of ENKTL tissue samples. (**B1** and **B2**) Negative immunohistochemical staining of adjacent nontumorous tissue. (**C1** and **C2**) Weak immunostaining of LMP1 in ENKTL tissue samples. (**D1** and **D2**) Strong immunostaining of LMP1 in ENKTL tissue samples. (**E1** and **E2**) Weak immunostaining of LMP2A in ENKTL tissue samples. (**F1** and **F2**) Strong immunostaining of LMP2A in ENKTL tissue samples. Original magnification × 200 in A1, B1, C1, D1, E1 and F1; ×400 in A2, B2, C2, D2, E2 and F2.

LMP1 and LMP2A protein levels in ENKTL were examined using immunohistochemistry. High LMP1 expression was detected in nine (56.25%) of 16 ENKTL tissues while high LMP2A expression was detected in seven (43.75%) of 16 ENKTL tissues (Table 1). Typically observed immunohistochemical staining patterns for LMP1 and LMP2A were shown in Figure 1. For LMP1, positive staining was mainly localized in the cell membrane and cytoplasm, while for LMP2A, positive staining was mostly located in the cytoplasm. No immunostaining of LMP1 and LMP2A was observed in adjacent nontumorous tissues.

### Correlation between LMP1 and LMP2A expression with clinicopathological parameters of ENKTL

The relationship between LMP1 and LMP2A protein levels with the clinicopathological parameters of 16 ENKTL patients was shown in Table 2. High LMP1 expression was associated with positive B symptoms (p = 0.012) and patients prognosis (0.049). No significant association was found between LMP1 expression and other clinical parameters, such as gender, age, Ann Arbor stage, LDH level, IPI or response to treatment. In contrast, high LMP2A expression was related to gender (p = 0.029) and patients prognosis (0.036). No statistical association was shown between LMP2A expression and other clinical parameters, including age, Ann Arbor stage, LDH level, B symptoms, IPI or response to treatment (Table 2).

**Table 2:** Association of LMP1 and LMP2A expression with clinical characteristics and selected biological markers of ENKTL

**Groups**	**No.**	**LMP1 expression**		**p value**	**LMP2A expression**		**p value**
		**Positive**	**Negative**		**Positive**	**Negative**	
Gender							
Male	13	7	6	0.687	4	9	0.029^*^
Female	3	2	1		3	0	
Age (years)							
≤40 y	5	3	2	0.838	3	2	0.377
>40 y	11	6	5		4	7	
Ann Arbor Stage							
I-II	14	7	7	0.182	6	8	0.849
III-IV	2	2	0		1	1	
LDH level							
High	5	3	2	0.838	2	3	0.838
Normal	11	6	5		5	6	
B symptoms							
Positive	8	7	1	0.012^*^	4	4	0.614
Negative	8	2	6		3	5	
IPI							
Low/Low-intermediate	11	5	6	0.197	4	7	0.377
High-intermediate/High	5	4	1		3	2	
Response to treatment							
Complete remission	7	4	3	0.493	4	3	0.319
Partial remission	8	5	3		2	6	
No response	1	0	1		1	0	
Prognosis							
Live	7	2	5	0.049^*^	1	6	0.036^*^
Dead	9	7	2		6	3	

LDH: lactate dehydrogenase; IPI: international prognostic index; LMP1: latent membrane protein 1; LMP2A: latent membrane protein 2A; * P < 0.05.

### Patients’ survival

**Figure 2:**
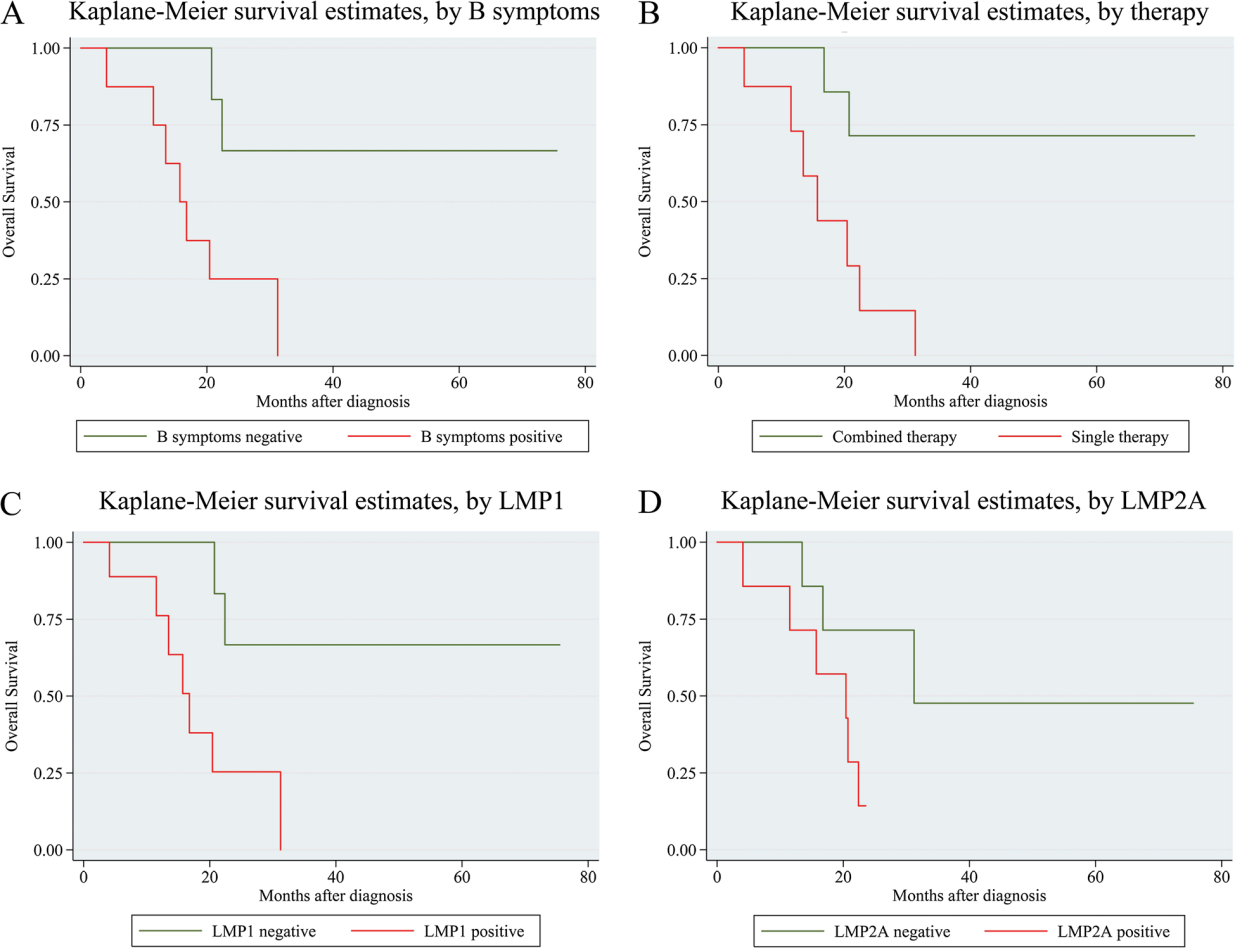
**Analysis of the survival of ENKTL patients by Kaplan-Meier method.** (**A**) Overall survival rate in patients with positive B symptoms (red line) was significantly lower than that in patients with negative B symptoms (green line). (**B**) Overall survival rate in patients with single therapy (red line) was significantly lower than that in patients with combined therapy (green line). (**C**) Overall survival rate in patients with positive LMP1 expression (red line) was significantly lower than that in patients with negative LMP1 expression (green line). (**D**) Overall survival rate in patients with positive LMP2A expression (red line) was significantly lower than that in patients with negative LMP2A expression (green line).

Kaplan-Meier analysis of 16 ENKTL patients showed that the survival rate was significantly lower in patients with positive B symptoms, subjected to single therapy, or with high LMP1 and LMP2A expression. Log-rank test showed that positive B symptoms, treatment by single therapy, and high expression of LMP1 and LMP2A were associated with decreased overall survival of ENKTL patients (P = 0.0180, P = 0.0082, P = 0.0201, P = 0.0487) (Table 3, Figure 2).

**Table 3:** Survival analyses of prognostic factors in ENKTL

**Variable**	**p value**
Gender	
Male versus Female	0.0658
Age (years)	
≤40y versus >40y	0.8679
Ann Arbor Stage	
Stage I, II versus Stage III, IV	0.4129
LDH level	
High versus Normal	0.7703
B symptoms	
Positive versus Negative	0.0180*
IPI	
L/LI versus HI/H	0.5418
Treatment	
Single therapy versus Combined therapy	0.0082*
LMP1 expression	
Positive versus Negative	0.0201*
LMP2A expression	
Positive versus Negative	0.0487*

LDH: lactate dehydrogenase; IPI: international prognostic index.

L: low risk; LI: low-intermiediate risk; HI: high-intermiediate risk.

* P < 0.05.

## Discussion

Most ENKTL cases show a uniform pattern of onset, typical pleomorphic morphology, and a similar NK cell immunophenotype. It is widely accepted that ENKTL is closely associated with EBV infection and abnormal expression of latent membrane protein products encoded by EBV [8-11]. Among these protein products, LMP1 is the most significant oncogenic protein which participates in the pathogenesis of EBV-associated lymphoma and is essential for EBV-induced B-cell transformation in vitro [12]. A variety of therapy strategies have been developed to target LMP1 or interfere with its downstream signal pathway, such as NF-κB, MAPK/MEK/ERK and JAK/STAT pathway, to inhibit tumor growth [23-25]. LMP2A is generated by alternative splicing and the expression of LMP2A in EBV-infected B cells may lead to the induction and maintenance of an activated, proliferative state that could ultimately result in the development of EBV-related lymphoma [20]. LMP2A has been utilized for targeted therapy and immunotherapy for lymphoma [26-28]. However, the role of LMP1 and LMP2A in ENKTL remains unclear.

In the present study, we examined the correlation of LMP1 and LMP2A expression with clinical characteristics of 16 ENKTL patients. The results showed that 56% (9/16) patients had positive LMP1 expression while 44% (7/16) had positive LMP2A expression. Moreover, LMP1 expression was obviously related to the presence of B symptoms and patients’ survival status, while LMP2A expression was related to gender and patients prognosis. It is interesting to notice that high expression of both proteins was correlated with poor prognosis. In addition, LMP2A expression appears to be related to gender. Although the relationship between LMP2A expression and gender was significant, we need to confirm it in later studies that enroll larger samples because only 3 female were enrolled in this study. Moreover, no statistical association was shown between LMP1 and LMP2A expression and other clinical parameters, including age, Ann Arbor stage, LDH level, IPI or response to treatment. Furthermore, Kaplan-Meier survival analysis showed that positive B symptoms, single therapy strategy and high expression of LMP1 and LMP2A could independently predict poor overall survival. These results are consistent with the regulatory effects of LMP1 and LMP2A on tumor cell growth, invasion, and metastasis reported in previous studies [14-17], and the poor prognosis of patients with high expression of LMP1 and LMP2A [3,29].

However, due to small sample size of this study, further studies that employ larger scale of clinical samples of ENKTL will be important to confirm our findings, which will provide the judgment on the values of LMP1 and LMP2A proteins for the diagnosis and prognosis of ENKTL.

## Conclusion

The expression of both LMP1 and LMP2A shows significant correlations with the prognosis of patients with ENKTL. LMP1 and LMP2A are novel prognostic markers of ENKTL.

## Competing interests

The authors declare that they have no competing interests.

## Authors’ contributions

YM and HL carried out all evaluation, and YM drafted the manuscript. HJZ, DWZ and LX carried out the immunohistochemistry experiments and helped perform the evaluation. QC helped draft the manuscript. YL and QDL collected clinical data and participated in the evaluation of the immunohistochemistry. JRX, LFX and RJC contributed to the conception and design of the study. All authors read and approved the final manuscript.
